# Estimation of genetic parameters for the implementation of selective breeding in commercial insect production

**DOI:** 10.1186/s12711-024-00894-7

**Published:** 2024-03-25

**Authors:** Laura Skrubbeltrang Hansen, Stine Frey Laursen, Simon Bahrndorff, Morten Kargo, Jesper Givskov Sørensen, Goutam Sahana, Hanne Marie Nielsen, Torsten Nygaard Kristensen

**Affiliations:** 1 Center for Quantitative Genetics and Genomics, Aarhus University, C F Møllers Allé 3, 8000 Aarhus, Denmark; 2 Department of Chemistry and Bioscience, Aalborg University, Fredrik Bajers Vej 7H, 9220 Aalborg, Denmark; 3 Department of Biology, Aarhus University, Ny Munkegade 116, 8000 Aarhus, Denmark

## Abstract

**Background:**

There is a burgeoning interest in using insects as a sustainable source of food and feed, particularly by capitalising on various waste materials and by-products that are typically considered of low value. Enhancing the commercial production of insects can be achieved through two main approaches: optimising environmental conditions and implementing selective breeding strategies. In order to successfully target desirable traits through selective breeding, having a thorough understanding of the genetic parameters pertaining to those traits is essential. In this study, a full-sib half-sib mating design was used to estimate variance components and heritabilities for larval size and survival at day seven of development, development time and survival from egg to adult, and to estimate correlations between these traits, within an outbred population of house flies (*Musca domestica*), using high-throughput phenotyping for data collection.

**Results:**

The results revealed low to intermediate heritabilities and positive genetic correlations between all traits except development time and survival to day seven of development and from egg to adulthood. Surprisingly, larval size at day seven exhibited a comparatively low heritability (0.10) in contrast to development time (0.25), a trait that is believed to have a stronger association with overall fitness. A decline in family numbers resulting from low mating success and high overall mortality reduced the amount of available data which resulted in large standard errors for the estimated parameters. Environmental factors made a substantial contribution to the phenotypic variation, which was overall high for all traits.

**Conclusions:**

There is potential for genetic improvement in all studied traits and estimates of genetic correlations indicate a partly shared genetic architecture among the traits. All estimates have large standard errors. Implementing high-throughput phenotyping is imperative for the estimation of genetic parameters in fast developing insects, and facilitates age synchronisation, which is vital in a breeding population. In spite of endeavours to minimise non-genetic sources of variation, all traits demonstrated substantial influences from environmental components. This emphasises the necessity of thorough attention to the experimental design before breeding is initiated in insect populations.

**Supplementary Information:**

The online version contains supplementary material available at 10.1186/s12711-024-00894-7.

## Background

Insects have been identified as a future important organic waste recycler and bioremediation agent [1–3]. On the industrial scale, insects are a novel source of human food [4], animal feed [5] and other value-added insect-derived products [6, 7]. This emerging agricultural sector represents a remarkable example of circular bioeconomy, where waste can be upcycled to high-value insect biomass contributing to long-term food security and environmental sustainability while minimising the exploitation of planetary resources and greenhouse gas emissions [8]. The proposal of entofarming as a part of sustainable agriculture has motivated the emergence of numerous industrialised facilities that are engaged in the domestication, production, and commercialisation of insects worldwide. As the number and scale of those productions increase, so does the necessity to optimise productivity.

Mass-rearing protocols for farmed insects have previously focused on optimisation through the manipulation of abiotic conditions, management and nutrition to ensure high survival and productivity [9–14], but further improvement could potentially be obtained by exploiting the genetic variation within and between populations [15]. Genetic optimisation through selective breeding is common practice in traditional crops and livestock, and more recently in aquaculture [16]. From a biological perspective, there is an enormous and untapped potential for genetic improvement in insects given the generally high levels of genetic variation in insect populations [17], short generation intervals and prolific reproduction that maintain large population sizes, thus enabling high selection intensity. However, the potential of these characteristics is understudied.

Selective breeding and its success rely entirely on the phenotypic superiority of an individual being attributed to its high genetic merit, which can be inherited through generations. However, identifying the potential for genetic improvement is not trivial and requires the segregation of phenotypic variance into additive genetic and environmental components. Quantifying the genetic parameters of the traits of interest is crucial for making rational breeding decisions, enhancing selection efficiency, prioritising traits to be improved, and optimising the selection strategy and breeding goal. In addition, obtaining information on genetic correlations between traits is necessary to avoid breeding for unfavourable outcomes, such as optimising an economically important trait at the expense of a fitness trait [15]. Although the idea of changing traits of interest through genetic improvement in insects is not novel [18], the estimation of genetic parameters in commercial insects has mainly been explored and used in honey bee (*Apis mellifera*) [19], silkworm (*Bombyx mori*) [20], and in the vinegar fly (*Drosophila melanogaster*) in laboratory settings [21]. Up to this point, selective breeding in commercial insect species has been based on simple phenotypic selection [22–24]. Sparse information is available on the genetic parameters of commercially important traits for farmed insects, and existing estimates are based on small sample size [25].

In livestock and crop breeding, the data used to estimate genetic parameters comprise phenotypic records from a large number of individuals or groups with known family relationships and/or genomic information. This is a typical framework, but obtaining similar information from insect populations poses particular challenges. The life-cycle, metabolism, morphology and reproduction of insects do not closely resemble those of other livestock species, and thus standard data registration procedures are not directly transferable. Commonly, insects have r-selected life history traits, including fast growth rate, short life span, and high fecundity [26], which have particular consequences when populations are mass bred in closed cycle captive breeding production systems. Possible consequences include loss of genetic diversity, decreasing fertility, low larval growth rates and fluctuating population viability [17], making it difficult to obtain sufficient data for accurate estimation of genetic parameters [27]. The holometabolous life cycle of commercial insect species prohibits the tracking of individuals in a population over time, since any physical tag would be shed at molting, pupation or eclosion (emergence from pupa). Re-identifying individuals repeatedly is essential for linking the individual to the traits observed at early and late life stages. Furthermore, the difficulties of sexing individuals at immature life stages complicate the assignment of phenotypic records that are obtained at the egg or larval stage to adult males and females [15]. Perhaps most importantly, the inability to continuously track individuals complicates the establishment and continuous maintenance of a pedigreed population, which is a fundamental requirement for obtaining phenotypic data for genetic parameter estimation. The relatively short life-cycle of most insects compared to other livestock adds further complexity, since the window for data recording and intervention is short and numerous individuals with synchronised life cycles have to be phenotyped simultaneously. Handling and phenotyping thousands of insects is labour intensive [28, 29] and this step is a major bottleneck. When phenotypic records from large populations are required, novel automated approaches need to be used to ensure accurate, fast, unbiased, and reproducible phenotyping across individuals, families, and generations.

The house fly (*Musca domestica*) is one of the species that has been commercialised for the production of protein intended for animal feed [6, 30, 31]. It has previously been extensively used in disease studies which can be attributed to its cosmopolitan nature and vector abilities. Recently, there has been a growing recognition of its potential as a bio converter, prompting comparisons with the black soldier fly (*Hermetia illucens*) in the context of intensive animal feed production [31]. In contrast to the black soldier fly, the house fly exhibits faster development, greater tolerance to variation in abiotic and periodically stressful conditions, and it has a more comprehensively studied biology and mating behaviour following decades of use as a model organism [31]. These attributes render it a fitting model system for experimental studies on selective breeding within the realm of commercial insects. The house fly has previously been the subject of quantitative genetics studies, mainly focused on the estimation of genetic parameters for morphometric and courtship traits, such as wing, head and body metrics and courtship repertoire elements, by mid- or single-parent–offspring covariance and regression analyses [27, 32]. One caveat of this approach is that upwards bias of the heritability estimates is introduced by covariance between offspring and mother due to maternal and other common environment effects, and regression of offspring on fathers is biased if the variance is not equal in the two sexes [33]. In addition, common environment effects complicate the estimation of genetic correlations between traits when using information on parents and offspring [34]. In contrast, when using appropriate models, the full-sib half-sib mating design allows for the separation of additive genetic, non-additive genetic and environmental (co)variance components, and thus unbiased estimates of heritabilities. However, this design puts high requirements on sample size.

The main objective of this study was to set up a large laboratory experiment with the aim of estimating genetic parameters for production and survival traits in a pedigreed house fly population. A typical aim of a breeding scheme is to optimise economic traits while decreasing time to harvest without sacrificing fitness [15]. Therefore, we included larval size, egg-to-adult development time and survival to larval and adult life stages as traits in this study. We used the full-sib half-sib design with isolated downscaled family rearing environments to maintain information on relatedness and used novel high-throughput phenotyping methods to obtain data for the quantitative genetic analyses. We established sib-groups to enable the estimation of traits measured at different life-stages. This design and results from this study comprise an important step towards implementation of genetic improvement programs in commercial insect production.

## Methods

### Base population

The population used in the experiment was an outbred population of the house fly established in June 2021 using flies collected from seven dairy cattle farms distributed across Denmark. Approximately 100 male and 100 female flies were collected from each cattle farm and set up for mating in farm-specific cages. A plastic cup containing larval medium and cotton soaked in a powdered milk solution was provided for egg laying and ~ 300 mg of eggs were collected from each of the seven populations. Offspring from each population were reared separately until the pupal stage, when 143 pupae from each population were distributed into each of 15 fly cages (W30xD30xH30 cm, BugDorm-1 Insect Rearing Cage, MegaView Science Co., Ltd., Taichung, Taiwan) yielding a total of ~ 1000 pupae per cage. This base population was maintained in the laboratory at a census size of ~ 15,000 individuals divided into three replicate subpopulations (population A, B and C) of ~ 5000 individuals, maintained in fly cages with ~ 1000 flies in each cage. In every generation, offspring from the five cages of one replicate subpopulation were intermixed at the pupal stage and randomly distributed into clean cages. Flies were reared under standard laboratory conditions at 23 °C, 40 to 60% relative humidity with a photoperiod of 12:12 h (light:dark) throughout their lifecycle. Larvae were reared on a standard laboratory larval medium (21.3% wheat bran, 10.7% alfalfa meal, 0.5% dry yeast, 0.8% malt and 66.7% tap water) and adults had access to water and a petri dish filled with equal parts of granulated sugar, icing sugar and powdered milk. The three replicate subpopulations were maintained in the laboratory for four generations before the initiation of the experiment.

### Full-sib half-sib mating design

**Fig. 1:**
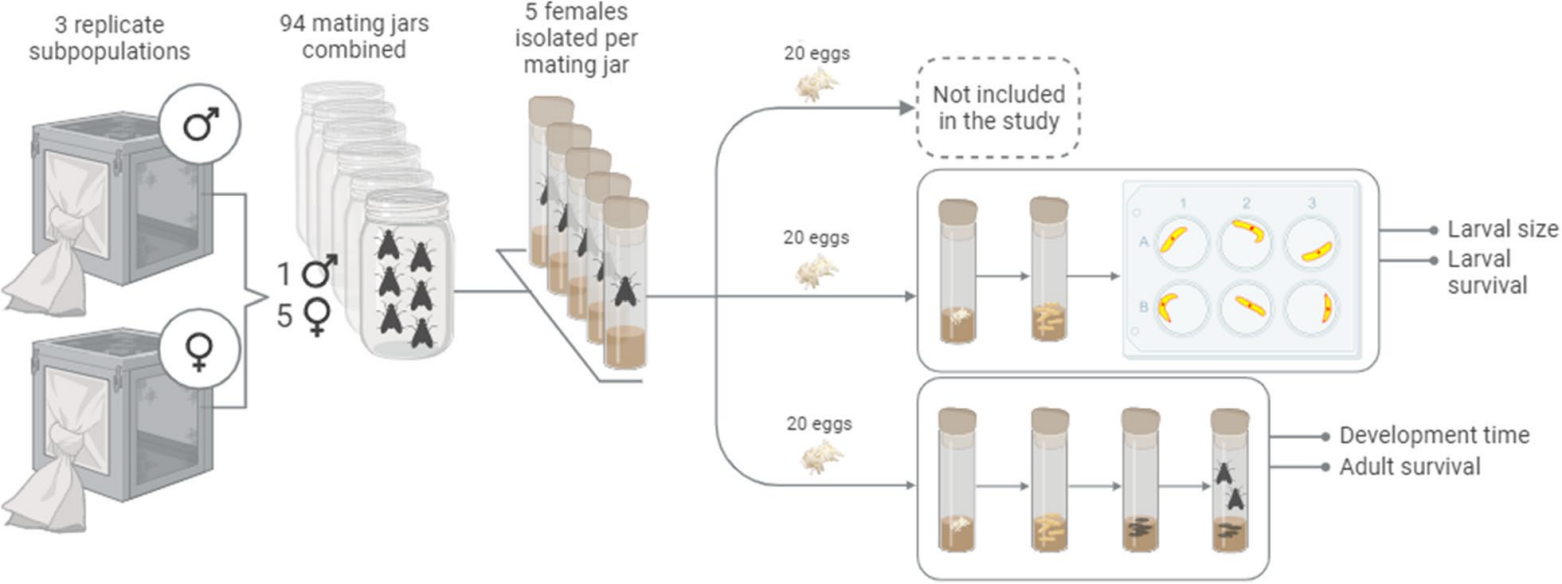
Illustration of the experimental design. Ninety-four male and 470 female house flies (*Musca domestica*) were joined in 94 mating jars (1:5 mating ratio). After 30 h, the female flies were isolated in oviposition vials. Eggs were collected every 12 h. Either zero, 20, 40 or 60 eggs were collected from a female depending on the size of her first egg clutch. Only eggs from the first oviposition event were used in the experiment. Two hundred and fifty-six females oviposited enough eggs for either one, two or three groups of full-siblings. Offspring were reared together in vials with maximum 20 offspring (density was dependent on mortality in the vials). One vial of full-siblings was reared until day seven, where larval traits were recorded. Another vial was reared until adult flies eclosed from the pupae and adult traits were recorded. The final vial with full-siblings was not included in the results of this study. Phenotypes were collected from 200 full-sib families. The illustration was created with BioRender.com

A nested paternal full-sib half-sib design was used when generating the families in this experiment (Fig. 1). The parent-generation was established by anesthetising and sorting newly emerged flies from the three replicate subpopulations every 24 h into sex-, age- and population replicate-specific fly cages. Virgin flies had access to granulated sugar, icing sugar, milk powder and water ad libitum. At the age of 10 d ± 12 h, one male and five female flies were sorted into glass jars (H: 11.5 cm, Ø: 6 cm) for mating. Each jar was sealed with a foam stopper fitted with a micro centrifuge tube (1.5 ml) with a 10% sugar solution. A second round of matings was established with 14-day old virgin flies from replicate populations A and B to achieve a sufficient experimental size (additional virgin flies were not available from replicate population C). A total of 94 mating jars were established when combining all replicate subpopulations (Table 1). Flies in the jars were allowed to mate for 30 h, where after all flies in each jar were anesthetised and females were sorted into oviposition vials (H: 9 cm, Ø: 2.5 cm) with 3 g fresh medium and kept in climate chambers at 23 °C, 60 to 70% relative humidity under a 12:12 h (light:dark) photoperiod. Oviposition vials were subsequently checked for eggs every 12 h until the female had either oviposited more than 20 eggs in one oviposition event or died. If no eggs were observed, the female was returned to the vial. If less than 20 eggs were found, females were transferred to a new oviposition vial with fresh medium. If more than 20 eggs were found, these were distributed into new vials with 10 g of fresh medium. Twenty eggs from each oviposition vial were designated for the measurement of larval traits, and 20 eggs were designated for the measurement of adult traits, ensuring that all traits were recorded on full siblings. Eggs were always collected from the same oviposition event and if a female oviposited enough eggs for the measurement of larval traits only, then no eggs were collected to measure adult traits. If more than 60 eggs were collected from a female, 20 eggs were designated for a third group of full siblings (not included in this study). All offspring were subsequently reared under the same conditions as the dams. The time from isolation until a female oviposited (time-to-oviposition, ± 12 h) was registered for all females. Due to the small numbers of eggs oviposited more than 24 h after isolation, the records were grouped in two levels (12 h and > 12 h). Since the eggs are vulnerable to damage and drying out during the handling process, the observer handling the eggs (observer) was registered for all eggs and thus, the effect of the observer could be tested on all traits recorded on the offspring. Observations from three out of six observers with the fewest records were combined under one observer label to avoid unbalanced group sizes in the dataset.

**Table 1:** Number of families for each of the three replicate populations

Replicate	Matings		Eggs		Offspring	
	Sires	Dams	Sires	Dams	Sires	Dams
A	36	180	34	94	28	74
B	39	195	36	107	29	83
C	19	95	19	55	17	43
Total	94	470	89	256^a^	74	200^b^

The number of families (sires = half-sib families, dams = full-sib families) for each step of the experiment; matings, successful ovipositions (eggs) and viable offspring (offspring) and for each replicate population (n = 3)

^a^2.86 dams per sire on average (not counting matings with zero egg-laying females)

^b^2.70 dams per sire on average (not counting matings with zero offspring-producing females)

### Phenotyping

Four traits were registered in this study; larval size, larval survival, egg-to-adult development time and egg-to-adult survival. Larval size was measured on all living offspring on the seventh day after the eggs had been collected using the automated size estimation procedure as described in Laursen et al. [28]. In short, full siblings from a vial were separated from the substrate and distributed individually into a transparent well-plate placed above a camera (25 frames per second, resolution: 1280 × 1024) (acA 1300-60gm GigE, Basler, Ahrensburg, Germany; lens: C-mount 4–8 mm, Computar, Tokyo, Japan) (see Additional file 1: Figure S1) and mean larval surface area was acquired for each larva with a single 60 s live imaging acquisition (base version of EthoVision XT 15.0.1418 with a custom JavaScript for size estimation, Noldus, Wageningen, The Netherlands). Larval survival was automatically obtained since all larvae with a size record were scored as survivors while those that did not survive until day seven were scored as dead.

Developmental time was observed every 12 h by checking vials for newly eclosed adults, which were removed from the vials and sexed under anaesthesia. Individual ID were assigned, and development time calculated for each adult (uncertainty is ± 6 h at egg collection and again at eclosion from pupa). All vials were continuously checked for four days post first eclosion. Individuals emerging as adults were scored as “survived” for adult survival. If none of the 20 offspring from a vial survived until phenotyping (either larval size or development time), and none of their full siblings had phenotypic records, all of the full siblings were scored as missing (“NA”) to avoid including eggs from non-fertilised females in the survival data. If some full siblings had phenotypic records, this was an indication that the female was indeed fertilised, and the missing offspring were scored as “dead” instead.

### Statistical analysis

Variance components were estimated for larval size, larval survival, development time and adult survival using univariate linear Gaussian sire models:

$${\mathbf{y}} = {\mathbf{X }}{\varvec{\upbeta}} + {\mathbf{Z}}_{1} {\mathbf{s}} + {\mathbf{Z}}_{2} {\mathbf{vial}} + {\mathbf{e}},$$

where $\mathbf{y}$ is a vector of phenotypes (larval and adult survival are binary); ${\varvec{\upbeta}}$ is a vector of fixed effects, which for larval traits included population replicate (three levels), observer nested within time-to-oviposition (eight levels) and dam age (two levels). For adult traits, the fixed effects included population replicate, observer (four levels) and sex (two levels, only included for development time). $\mathbf{s}$ is the vector of random sire effects, $\mathbf{s}\sim N({\mathbf{0}},\mathbf{I}{\sigma }_{s}^{2})$ and sires were assumed unrelated; $\mathbf{v}\mathbf{i}\mathbf{a}\mathbf{l}$ is a vector of random rearing vial effects, $\mathbf{v}\mathbf{i}\mathbf{a}\mathbf{l}\sim N({\mathbf{0}},\mathbf{I}{\sigma }_{vial}^{2})$; $\mathbf{e}$ is the vector of random residuals, $\mathbf{e}\sim N({\mathbf{0}},\mathbf{I}{\sigma }_{e}^{2})$; $\mathbf{I}$ is an identity matrix and $\mathbf{X}$, ${\mathbf{Z}}_{1}$ and ${\mathbf{Z}}_{2}$ are the corresponding incidence matrices. Since offspring reared in the same vial came from the same dam, the vial effect also included the effect of the dam. Sire models were used since the dataset comprised phenotypic records from a single generation of individuals, and the heritability (${{\text{h}}}^{2}$) for each trait was estimated as ${{\text{h}}}^{2}= \frac{{4\upsigma }_{{\text{s}}}^{2}}{{\upsigma }_{{\text{s}}}^{2}+{{\upsigma }_{{\text{vial}}}^{2}+\upsigma }_{{\text{e}}}^{2}}$. Variance components were estimated using restricted maximum likelihood with the average information algorithm (AI-REML) implemented in the DMUAI module of the DMU software package [35]. Standard errors of the heritability estimates were calculated using the delta method [34]. To estimate a confidence interval for the heritability estimates, variance components were re-estimated using random re-samplings of 90% of the phenotypic records (100 iterations). Resampling was done without replacement.

**Fig. 2:**
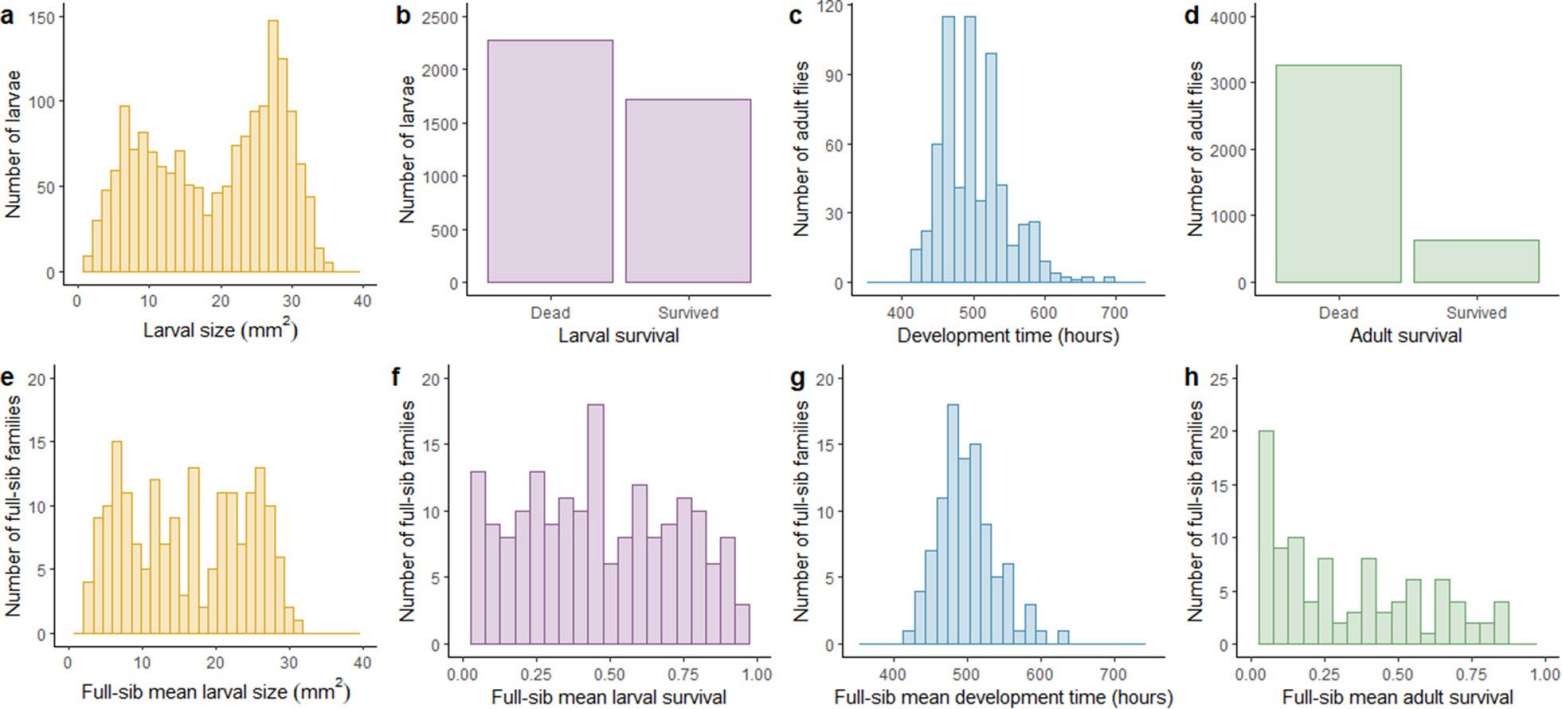
Individual and average phenotype distributions. Distributions for larval size (**a** and **e**) and survival (**b** and** f**), development time (**c** and **g**) and adult survival (**d** and** h**). Panels in the top row are distributions based on individual records and panels in the bottom row are based on family averages (n = 1723, 4000, 630 and 3900 for the individual records and 184, 200, 96 and 195 for the family average records of larval size, larval survival, development time and adult survival, respectively)

Larval size data had a bimodal distribution (Fig. 2) possibly caused by an unknown factor that strongly affected the trait. The estimated variance components differed when the two peaks were analysed separately compared to when the full dataset was analysed together (see Additional file 2: Table S1 and Additional file 3: Table S2). Therefore, larval size records were normalised using quantile normalisation where all records were standardised according to the fixed effect of an unknown binary variable splitting the two larval size peaks (cut-off at 18.999 mm^2^ based on visual inspection of the phenotype distribution). The standardised records were analysed using the same linear Gaussian sire model as the raw records. In addition, the heritability of the binary larval size trait (being “small” or “large”) was estimated using the same linear Gaussian sire model as the raw and the standardised records.

Bivariate linear Gaussian sire models were used for the analysis of covariance between all four traits. Since larval size and development time were inevitably only recorded for surviving offspring, the correlations between all pairwise trait combinations were estimated using full-sib mean phenotypes. The bivariate models contained the same fixed effects as the univariate models for each trait, and sire as a random effect. In the bivariate model for the average larval size and development time, a weight according to the number of full-sib records used to compute the averages was applied to account for heterogeneous residual variances due to different family sizes. A weight was not applied to survival traits, since the family size was constant at 20 individuals. AI-REML in DMU was used to estimate the genetic and phenotypic correlations [35], and the variance component estimates from univariate models were used as starting values for the algorithm (starting values for covariances were set to zero). For larval size and development time, the genetic correlation was also estimated using individual-level phenotypic records. The model used for this estimation contained the same fixed effects as the univariate models used for the estimation of the variance components for the two traits. Given the anticipated covariance between full siblings due to their shared mother, the dam was added as a random effect. The vial effect was excluded from the model due to its complete confounding with the dam. The covariance between the residuals was restricted to zero, since the two traits were measured on different offspring. For this reason, the phenotypic correlation could not be estimated between larval size and development time when using the individual-level records. The standard errors of the genetic and phenotypic correlations were calculated using the delta method [34]. All additional statistical analyses were performed in R v.4.2.0 [36].

## Results

### Mating and oviposition success

In total, 470 mating pairs were set up in the experiment. From these, 256 dams oviposited and 200 of those had offspring with phenotypic records (Table 1). Larval traits were collected from 200 full-sib families, of which 184 produced offspring with records for larval size. The adult traits were collected from 195 full-sib families from which 96 had records of egg-to-adult development time. There were no survivors in 16 vials for the larval traits and 99 vials for the adult traits, respectively. However, since their full siblings had records for traits at the other life stage, or for the traits not included in this study, they were all included in the dataset as “dead”. Fifty-four percent of the dams produced eggs and 95% of the sires mated with egg-laying females. Seventy-nine percent of the sires and 43% of the dams had offspring, which were assessed for larval/adult traits. The number of egg-producing and offspring-producing females per sire is in Additional file 4: Figure S2. Eleven of 89 sires produced eggs with only one dam, 16 of 73 sires produced offspring with only one dam which were phenotyped at the larval stage, and 27 of 52 sires produced offspring with only one dam which were phenotyped at the adult stage.

### Summary statistics

Phenotypes from 7900 offspring were collected for larval or adult traits in this study (Table 2). Larval trait records were collected from 4000 individuals, and adult trait records from 3900 individuals. The survival from egg to 7-day-old larvae was 43%, and thus larval size was collected for the 1723 surviving larvae. The survival to adulthood was only 16%, so egg-to-adult development time was collected for 630 adult flies. The number of larvae measured in each family ranged from 1 to 20 surviving individuals, with egg-to-adult development time ranging from 1 to 17 adult offspring.

**Table 2:** Summary statistics for larval and adult traits

	N	Sires	Dams	Mean	SD	Min	Max
Larval size (mm^2^)	1723	73	184	18.97	9.11	1.30	35.13
Larval survival	4000	74	200	0.43	0.50	0.0	1.0
Development time (h)	630	52	96	500.29	45.17	422.00	686.00
Adult survival	3900	74	195	0.16	0.37	0.0	1.0

Number of observations (N), half-sib families (sires), full-sib families (dams), mean, standard deviation (SD), minimum (min) and maximum (max) for each of the four traits. The total number of offspring with records was 7900, which is the sum of the offspring with a survival record at the larval or adult stage. Larval size and development time were recorded for the surviving proportion of the offspring

### Trait distributions

We observed large variation in larval size measured at an age of seven days (Fig. 2a). When investigating the mean size of full-sib larvae from each vial, the size range was still large and the bimodal distribution, which was also observed with the individual records could still be observed, although less clearly (Fig. 2e). The total range of family average records was comparable to the total range observed for individual records. Likewise, there was a large variation in development time (Fig. 2c) with a heavy right tail towards longer development times. This variation was also evident for the vial mean development time (Fig. 2g). Which developmental stage caused the difference between slow and fast developing individuals is unknown, since the vials were not monitored during the period between egg-collection and adult eclosion. Most flies emerged during daytime, resulting in a diurnal alternation in number of development time records. The mean family larval survival was uniformly distributed from survival proportion 0 to 100%, with a peak in survival just below 50% and a slight left skew towards lower survival (Fig. 2f). The mean survival to adulthood was heavily skewed towards low vial averages, although a large variation between vials was still observed (Fig. 2h).

### Systematic effects

We observed several systematic effects on the recorded traits (see Additional file 5: Table S3). The larvae produced by the older dams were smaller and had a lower survival than those from younger dams by 9.5 mm^2^ and 0.26 percentage points, respectively. Generally, a delay in oviposition from the time of mating resulted in smaller larvae and lower survival, with some differences between observers. The observer effect originates from the handling of the eggs and indicates a difference in handling especially between observer 3 and the other observers. However, it is important to note the difference in number of records between observer 3 and the other observers. The substantially smaller number of eggs handled by observer 3 could contribute to the effects of oviposition delay being contradictory between observers simply by chance. In addition, all observers, except for observer 3, handled eggs which were oviposited more than 24 h after female isolation (up to 72 h). Thus, the negative effects of delayed oviposition on size and survival are naturally stronger for observer groups 1, 2 and 4. The effect of dam age and time-to-oviposition could not be included in the analysis of adult traits since almost no adults emerged from the group of old dams and oviposition time longer than 12 h. These results clearly indicate that the age of the dam and the slow oviposition had a negative impact on the offspring. In addition, male offspring eclosed from the pupa earlier than female offspring by 10 h. There were minor differences between the three replicate populations for all traits, with population C tending to have smaller larvae, a longer development time and a lower larval and adult survival.

### Genetic parameters

The larval traits generally have low heritability estimates with large standard errors (Table 3). It is evident from the large resampling confidence intervals that estimates are sensitive to variation in the randomly sampled phenotypic records. The vial effect, which is the effect of common environment and maternal effects, is several magnitudes larger than the sire effect for all traits. The sire effect is confounded with the vial effect in cases where a sire successfully reproduced with only one female. The number of families was not sufficiently large to estimate variance components, separately, for each replicate population.

**Table 3:** Genetic parameters for larval and adult traits

	N	Sires	Dams	${\sigma }_{s}^{2}$	${\sigma }_{vial}^{2}$	${\sigma }_{e}^{2}$	h^2^ (SE)	CI of h^2^
Larval size (mm^2^)	1723	73	184	1.64	32.18	30.16	0.10 (0.21)	[0.034;0.17]
Larval size (quantile normalised)	1723	73	184	0.021	0.19	0.73	0.09 (0.11)	[0.029;0.14]
Larval size (binary)	1723	73	184	0.0055	0.087	0.11	0.11 (0.19)	[0.05;0.18]
Larval survival	4000	74	200	0.0042	0.044	0.17	0.075 (0.081)	[0.046;0.10]
Development time (hours)	630	52	96	120.11	1140.03	693.21	0.25 (0.44)	[0.078;0.44]
Adult survival	3900	74	195	0.014	0.040	0.081	0.41 (0.15)	[0.37;0.44]

The number of observations (N), half-sib families (sires), full-sib families (dams), sire (${\upsigma }_{{\text{s}}}^{2}$), vial (${\upsigma }_{{\text{vial}}}^{2}$) and residual (${\upsigma }_{{\text{e}}}^{2}$) variance components, heritability estimates (h^2^) with standard errors (SE) and confidence intervals of heritability estimates (CI of h^2^) for larval size, quantile normalised larval size, larval survival, development time and adult survival across all three replicate populations

Larval size was strongly positively correlated with larval survival, both genetically (0.68) and phenotypically (0.56) (Table 4). The genetic correlation between larval size and development time was positive, both when estimating the correlation using family averages (0.47) and individual-level records (0.60 (1.40), not shown in Table 4), while the phenotypic correlation was close to zero (− 0.05). Development time was moderately negatively genetically correlated with larval survival (− 0.21), and strongly genetically correlated with adult survival (− 0.67) although both phenotypic correlations were positive (0.37 and 0.34, respectively). All genetic correlation estimates had large standard errors. The bivariate models used to analyse the covariance between adult survival and larval size and survival did not reach convergence.

**Table 4:** Genetic and phenotypic correlations

	Larval size	Larval survival	Development time	Adult survival
Larval size	–	0.56 (0.17)	− 0.05 (0.17)	NC
Larval survival	0.68 (0.59)	–	0.37 (0.18)	NC
Development time	0.47 (0.47)	− 0.21 (0.84)	–	0.34 (0.22)
Adult survival	NC	NC	− 0.67 (0.55)	–

Values above the diagonal are phenotypic correlations (± SE), below the diagonal are genetic correlations (± SE) between pairwise combinations of larval size and survival, egg-to-adult development time and egg-to-adult survival. NC indicates that the algorithm did not converge

## Discussion

As insect farming has gained renewed attention due to its efficacy in bio conversion and as a source of livestock feed [14, 37–39] the demand for genetic improvement targeting desirable traits has become increasingly evident. In our experiment, we present a novel experimental design using the house fly as a model species for genetic evaluations in commercial insects. We established 470 individual mating pairs nested within 94 half-sib families, with the aim to obtain accurate estimates of genetic parameters [40], and we successfully collected phenotypic records for larval and adult traits from offspring produced in 96 to 200 full-sib families. Heritability estimates were all above zero, although with substantial standard errors, and genetic parameters indicated sufficient genetic variation in all studied traits to enable genetic improvement through selective breeding. However, it was evident that non-genetic factors made substantial contributions to the observed phenotypic variation, indicating the need to improve the experimental design and identify these sources of variation. Multi-trait analyses revealed both favourable and unfavourable correlations among different traits. A detailed discussion of our findings is presented below.

### Variance components and heritability

The estimates of residual and vial variances were generally high for all traits relative to the additive genetic variances, revealing considerable environmental influence on the traits. Heritability estimates were low for larval traits when using both raw and quantile normalised data, whereas the estimates were moderate for adult traits. Most quantitative genetics studies on the house fly have been performed on adult morphometric traits several decades ago [22, 27, 41, 42] and to the best of our knowledge, there is no recent study reporting estimates of genetic parameters for production traits in this species. From theory, low narrow-sense heritability was expected for life-history traits relative to morphological traits, since traits associated with fitness generally exhibit a lower additive genetic variance and are influenced by more developmental and environmental noise than morphological traits [43]. This might not be true for ectotherm larval morphology, which could be influenced by many developmental pathways, as well as being highly sensitive to environmental variation [44–46]. The influence of environment on phenotype may decrease as the flies develop, if juvenile life-stages are especially prone to environmental disturbances [47, 48]. This could result in higher heritability estimates for adult compared to larval traits, but with standard errors simultaneously increasing due to reduced sample size for adult traits. A key insight from this study underscores the need for a careful design of experimental protocols that are aimed at estimating genetic parameters in insect populations. Such protocols should facilitate the quantification of and correction for environmental influences that stem from scale-related factors or other non-genetic sources. This becomes especially important as selection in commercial insect populations will likely be based on family information, where the covariance between members of a full-sib group can be severely inflated by shared environment. In addition, our study revealed that a substantial number of males did not successfully reproduce with more than one dam. In those cases where no half-sib information is available, the additive genetic effect is estimated from full-sib information only and is thus confounded by both non-additive genetic and environmental effects. This bias increases with developmental stage as the variance components estimated for egg-to-adult development time rely on full-sib information more often than for larval size. Such a bias can result in an inflation of the heritability estimates, especially if common environment effects strongly affect the full-sib groups. Thus, it is essential to optimise mating and reproduction to minimise such a bias in future studies and in a production setting.

### Genetic correlations

A positive genetic correlation was observed between larval size and development time. This would be an unfavourable correlation in a production setting that aims at increasing yield and decreasing time-to-harvest. The positive genetic correlation between larval size and development time is in agreement with life-history theory [49] and with previous studies on *D. melanogaster* [50]. For *D. melanogaster*, it has been reported that the correlation between adult body size and development time was positive when using wild flies, but negative when using a laboratory cross [51]. This demonstrates a potential trade-off, where larvae accumulate body mass at the expense of early pupation and eclosion success. Larraín and Salas [45] demonstrated that high quality organic substrates produce large larvae, fast development and high survival in house flies. Such a result highlights the importance of estimating genetic correlations before initiating a selection scheme, to unveil unfavourable correlations that might be masked by the environment. In addition, it emphasises the importance of employing a multi-trait breeding program to prevent adverse selection responses in traits of interest, which could occur as a result of unfavourable correlations between traits in the breeding objective. On the contrary, large size and fast development were both positively correlated with high survival in our study. For future implementation of genetic improvement programs, this is promising, since increasing yield and decreasing time to harvest would not compromise survival, although the correlation estimates might differ in a less controlled production setting. The opposite result has been reported for *D. melanogaster*, where selection for large (adult) size resulted in a decline in larval viability [50]. The genetic correlations estimated in our study are not straightforward to interpret and seemingly point in different directions in terms of the correlation between development time, larval size and larval survival. It is important to note that correlation estimates from our study should be interpreted with caution due to their large standard errors. Obtaining accurate estimates of genetic correlations demands a substantially larger number of observations compared to the estimation of variance components, especially for traits with a low heritability [52]. The number of families and phenotypic records obtained in this study would be sufficient to estimate genetic correlations if the individual records could be used. Given the difficulty associated with tracking individuals from juvenile to adult life stages, the use of family averages for estimation of genetic covariance and, eventually, for genetic evaluations in breeding schemes, is expected to be increasingly adopted in insect breeding in the future. To maximise statistical power while ensuring feasibility, the number of half-sib families should be maximised rather than the number of offspring per family (see [53] for a discussion on statistical power and experimental design for the estimation of genetic correlations). This recommendation is contingent upon maintaining the ability to obtain a reliable phenotypic average from a full-sib group.

### Mating and oviposition

To date, there is little information available on house fly re-mating ability. Females generally do not re-mate, while male re-mating has been observed [54]. Our study confirms that male house flies can successfully reproduce with five females, although a majority of males did not successfully fertilise all available females in the allotted mating period. The results further indicate that a proportion of females oviposited without being fertilised, since 56 of the 256 egg-laying females did not produce any live progeny. Virgin oviposition in dipterans is not uncommon [55] and it has been suggested that the lifetime virgin egg production in the house fly is approximately 40% that of mated females [56]. These studies provide evidence that ovipositional activation occurs almost immediately after mating, whereas virgin females experience a delay in egg deposition. In this case, restricting egg collection to a short window following mating, and simultaneously lowering the ratio of females to males, could reduce the proportion of unsuccessful mating and oviposition.

### Phenotypic variation

Sample size was drastically reduced due to low survival, especially from egg to adulthood. Out of the 3900 eggs collected from 195 females for the recording of adult traits, only 16% of the offspring emerged as adults. Reported survival proportions from egg or first-instar larvae to adulthood are within the range from 43 to 80% [57, 58], although mortality depends highly on the rearing substrate [45]. Low mortality is expected from offspring produced on the standard laboratory substrate used in this study and thus the low survival in our study is somewhat surprising. However, it may be related to the fact that individual larvae or flies cannot be tracked in a larger rearing system over time, as we had to isolate full-sib families, and thus smaller containers were required to keep track of pedigree information. Unfavourable conditions caused by such downscaling, such as drying and encrusting of the rearing substrate [45], low temperature [59], accumulation of ammonia in the small vials [60], reduced microbial activity [61] or insufficient processing of the substrate through ‘social digestion’ [62] could cause retarded growth and reduced survival in dipterans. The average development time is observed to be longer in our study compared to other findings in the literature [45, 58, 60], which is an indicator of suboptimal rearing conditions. Furthermore, it has been suggested that social isolation can affect non-behavioural phenotypes such as development and mortality [63]. Bryant and Meffert [64] report a loss of experimental size when attempting to track relatedness by grouping individual house flies in smaller containers, due to low viability (50 families intended, 21 to 38 successful). The substantial variation in survival between vials likely introduces cryptic patterns of density effects on larval development. Since density is neither controlled nor monitored after the egg stage, it complicates the inclusion of density effects in the analyses presented in our study. However, since the variation ranges from 1 to 20 for larval records and from 1 to 17 for adult records per vial, density inevitably impacted the measured traits. The positive phenotypic correlation between larval size and larval survival, and between development time and adult survival, can be interpreted as a density effect and indicates moderate to strong effects of density on the measured continuous traits. Upscaling is often reported as a major challenge in commercial insect production, but our findings reveal that downscaling also remains a major challenge for the establishment of pedigreed insect populations. Possible solutions include increasing the family size to create more robust rearing environments and reduce mortality. Performing density control throughout development could reduce variation in density between rearing environments but might introduce unwanted effects of frequent handling and would be highly labour intensive. While it is crucial to optimise the family-based setup with spatially downscaled environments without compromising survival or fitness of the population, it is also imperative to exercise caution to prevent genotype by environment interactions from obscuring the transferability of genetic improvement to the production environment.

Using the automated high-throughput phenotyping setup for larval size measurements [28] allowed for a highly synchronised experimental design and facilitated fast phenotyping of offspring with minimised age differences. This synchronisation of age is crucial for selective breeding, where selection candidates are required to be compatible for mating. In spite of the small variation in age, the phenotypic variation in larval and adult traits was large, and this could not be explained exclusively by systematic effects such as sex, dam age, time-to-oviposition or observer effects. The bimodal larval size distribution could be a consequence of sexual dimorphism in body size since it was impossible to distinguish between male and female larvae. In many insect species females are larger than males [65], and in *D. melanogaster*, this is already observed in late instar larvae [46]. However, Siomava et al. [46] did not observe differences in pupae size between male and female house flies. In addition, it seems unlikely that the bimodality in larval size would remain evident in the family averages, if it was exclusively a result of sexual dimorphism. A high degree of heterogeneity in vial conditions constitutes another probable explanation, where vials with poor conditions produce low survival and small larvae, and vice versa for vials with favourable environmental conditions. In this case, density-related differences between vials could contribute to the bimodality observed in the larval size distribution. However, 168 of the 184 families with records for larval size are represented in the “small size” peak, whereas only 111 families are represented in the “large size” peak (see Additional file 2: Table S1). If density differences between the vials was the source of the bimodality in larval size, we expect vials to hold mainly larvae below or above the threshold separating the two peaks in size, since all larvae in one vial experienced the same density conditions.

### Future considerations

In this study, the estimation of genetic parameters was based on a single generation of phenotypic records from full- and half-sibs. For this reason, sire models were used for the estimation of genetic parameters. In a scenario where phenotypic records from multiple generations of individuals with many different genetic relationships are available, the animal model is superior for the estimation of genetic parameters and genetic evaluation. The depth and completion of a pedigree are important for the prediction of genetic values of individuals or families, since additive effects are inherited across generations and integrating phenotypes of relatives through the link of ancestors will improve accuracy of predictions in the current generation [66]. Maximising the number of individuals with phenotypic records in the current generation while including diverse types of family relationships when constructing the pedigree will increase the reliability of variance component estimates, especially for traits characterised by a low heritability [33]. Reassessing the genetic parameter estimates becomes necessary upon the acquisition of a deeper pedigree, for example in the context of a commercial breeding setup and in different environments. It is important to emphasise that our estimates are only valid for the population and environment investigated. The variance components depend highly on the environmental conditions and will differ in a production environment. We present a proof-of-concept for obtaining genetic parameter estimates on the house fly, but transferability of the estimates to other rearing systems or species should be done with caution.

Implementation of genetic selection strategies in farmed insects will be successful only when the rearing requirements, mating behaviour, reproduction and inheritance patterns of economically important traits are clearly understood. Therefore, estimating genetic parameters is critical for insect breeding research and for implementing genetic improvement programs in the industry. In our study, larval traits exhibited a low heritability and thus the rate of genetic improvement is expected to be slow for those traits if selection is based exclusively on phenotypes. In the case where additive variance constitutes a small proportion of the total phenotypic variance, the performance of an individual will not lead to accurate estimates of breeding values that are used to identify the parents of the next generation. However, this is not decisive for whether to include the traits in a breeding program, as the economic circumstances might justify the attention to larval traits. If larval size and survival are of substantial economic value, improvement through optimised environmental conditions in the industrial setting could increase the heritability, and thus ensure faster genetic improvement. Whether the genetic potential unfolds more or less under optimal conditions would need further investigation. In addition, advanced breeding programs using pedigree or genomic information could improve the selection efficiency for these low-heritability traits. A natural next step in this area is to perform the necessary economic evaluations to prioritise traits in a breeding goal according to their economic values, and also identify which selection strategy to apply. Due to the limitations associated with individual tracking of insects, evaluations and selection at the family level are likely the future of insect breeding. Given that rearing full-sibs together represents the most genetically diverse group configuration possible while retaining knowledge on the pedigree, investigating the sizes of full-sib groups and incorporating considerations of shared environmental effects offers an opportunity for improving the family-based design, which is sensitive to bias from shared maternal effects, common environment and group size. Maintaining an entire production population of insects in family-specific rearing environments is unfeasible and will introduce massive variation. Establishing a smaller breeding nucleus from which genetic material can be disseminated is a more realistic breeding structure and would enable the implementation of advanced and labour-intensive breeding programs. Finally, it has been demonstrated that insect populations of commercial interest are susceptible to the detrimental consequences of inbreeding depression [17], which can hinder the advancements achieved through selection. It is therefore imperative to investigate strategies that maximise progress while concurrently mitigating the risks associated with inbreeding in a family selection scheme.

## Conclusions

Entofarming is a crucial stride towards the implementation of sustainable agricultural practices and the integration of genetic selection in farmed insects holds the potential to enhance the associated benefits further. The estimation of genetic parameters serves as an initial and indispensable step in facilitating selective breeding efforts for commercial insect species and this study provides important insights on this subject. Estimated heritabilities indicated that a portion of the variation observed in the four investigated traits can be attributed to additive genetic variance. The estimate for development time indicated a surprisingly high heritability in relation to larval size although the confidence intervals for the estimates largely overlap. We further observed positive genetic correlations between trait combinations involving larval size, but negative for those involving survival traits and development time, although these estimates were accompanied by large standard errors. The use of a full-sib half-sib design proves to be suitable for isolating additive genetic effects in house flies, and potentially in other commercially relevant insect species as well. However, it is essential to adjust the male-to-female ratio in accordance with the mating behaviour of the species under consideration. Using technological advances when acquiring phenotypic data from fast-developing organisms is essential to reach a sufficient number of phenotypic records, and this approach also facilitates age synchronisation among selection candidates to enhance mating compatibility. Leveraging family phenotype averages from full-sibs reared together offers the advantage of maintaining a pedigreed population without requiring individual-level tracking throughout time, but the risk of inflated covariance due to shared maternal and common environment effects should be considered and accounted for, if possible. Nevertheless, the standardisation and optimisation of the production environment for individuals, families and populations, including challenges associated with downscaling, remain significant challenges that warrant primary focus prior to launching breeding endeavours.

### Supplementary Information

**Additional file 1: Figure S1.** Phenotyping setup for larval size measurements. Picture of the automated phenotyping setup for larval size measurements.

**Additional file 2: Table S1.** Summary statistics for bimodal distribution analysis. Summary statistics for the separate analysis of small and large larvae.

**Additional file 3: Table S2.** Genetic parameters for bimodal distribution analysis. Genetic parameters for the separate analysis of small and large larvae.

**Additional file 4: Figure S2.** Mating success. Figure illustrating the number of females each male house fly reproduced with.

**Additional file 5: Table S3.** Systematic effects. Table of fixed effect estimates from univariate Gaussian models for each of the analysed traits.

## Data Availability

The dataset supporting the conclusions of this article is available in the Dryad repository, 10.5061/dryad.kwh70rzc5.
