# Supplementary material for: Estimation of genetic parameters for the implementation of selective breeding in commercial insect production
Source: Genet Sel Evol. 2024 Mar 25;56:21. doi: 10.1186/s12711-024-00894-7 (PMC10962107; doi:10.1186/s12711-024-00894-7)
Supplement: Supplementary file 1 — Additional file 1: Figure S1. Phenotyping setup for larval size measurements. Picture of the automated phenotyping setup for larval size measurements. [file 12711_2024_894_MOESM1_ESM.docx]

**Additional file 1 Figure S1: Phenotyping setup for larval size measurements**

**
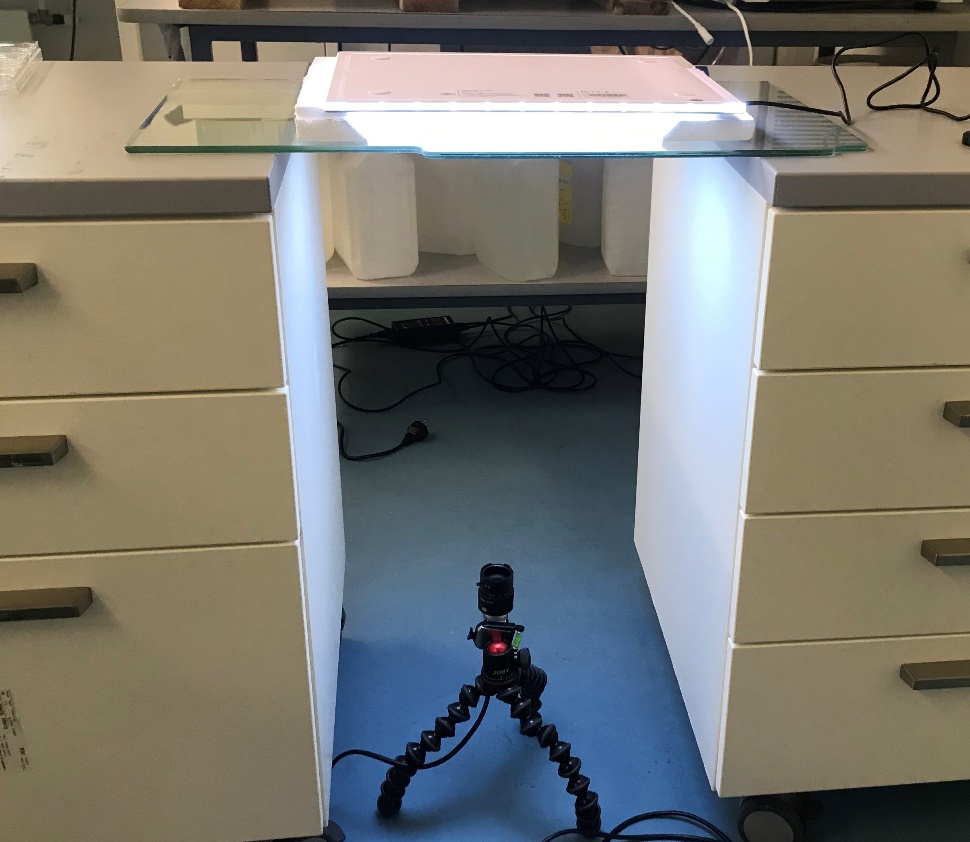
**

**Phenotyping setup for larval size measurements.** The camera was placed underneath a glass plate with a light source above. Larvae were placed in clear plastic well plates which were inserted between the light and glass plate for the recording of larval surface area from below.
