# Supplementary material for: Estimation of genetic parameters for the implementation of selective breeding in commercial insect production
Source: Genet Sel Evol. 2024 Mar 25;56:21. doi: 10.1186/s12711-024-00894-7 (PMC10962107; doi:10.1186/s12711-024-00894-7)
Supplement: Supplementary file 2 — Additional file 2: Table S1. Summary statistics for bimodal distribution analysis. Summary statistics for the separate analysis of small and large larvae. [file 12711_2024_894_MOESM2_ESM.docx]

**Additional file 2 Table S1: Summary statistics for bimodal distribution analysis**

**Summary statistics for small and large larvae**

|  | N | Sires | Dams | Mean | SD | Min | Max |
| --- | --- | --- | --- | --- | --- | --- | --- |
| Larval size (< 18.999 mm^2^) | 795 | 71 | 168 | 10.13 | 4.41 | 1.30 | 18.98 |
| Larval size (> 18.999 mm^2^) | 928 | 53 | 111 | 26.55 | 3.60 | 19.02 | 35.13 |

Number of observations (N), half-sib families (sires), full-sib families (dams), means, standard deviation (SD) minimum (min) and maximum (max) larval size of small (< 18.999 mm^2^) and large (> 18.999 mm^2^) larvae. The total number of offspring with larval size records were 1723, which is the sum of small and large larvae. The threshold of 18.999 mm^2^ was decided based on visual inspection of the size distribution.
