# Supplementary material for: Estimation of genetic parameters for the implementation of selective breeding in commercial insect production
Source: Genet Sel Evol. 2024 Mar 25;56:21. doi: 10.1186/s12711-024-00894-7 (PMC10962107; doi:10.1186/s12711-024-00894-7)
Supplement: Supplementary file 3 — Additional file 3: Table S2. Genetic parameters for bimodal distribution analysis. Genetic parameters for the separate analysis of small and large larvae. [file 12711_2024_894_MOESM3_ESM.docx]

**Additional file 3 Table S2: Genetic parameters for bimodal distribution analysis**

**Genetic parameters for small and large larvae**

|  | N | Sires | Dams | $\boldsymbol{\sigma}_{\boldsymbol{s}}^{\boldsymbol{2}}$ | $\boldsymbol{\sigma}_{\boldsymbol{vial}}^{\boldsymbol{2}}$ | $\boldsymbol{\sigma}_{\boldsymbol{e}}^{\boldsymbol{2}}$ | h^2^ | SE | CI of h^2^ |
| --- | --- | --- | --- | --- | --- | --- | --- | --- | --- |
| Larval size  (< 18.999 mm^2^) | 795 | 71 | 168 | 0.90 | 5.79 | 9.71 | 0.22 | 0.22 | [0.099;0.33] |
| Larval size  (> 18.999 mm^2^) | 928 | 53 | 111 | 2.41 | 3.03 | 7.98 | 0.72 | 0.29 | [0.56;0.83] |

The number of observations (N), half-sib families (sires), full-sib families (dams), sire ($\sigma_{s}^{2}$), vial ($\sigma_{\mathrm{vial}}^{2}$) and residual ($\sigma_{e}^{2}$) variance components, heritability estimates (h^2^) with standard errors (SE) and confidence intervals of heritability estimates (CI of h^2^) for small (< 18.999 mm^2^) and large (> 18.999 mm^2^) larvae across all three replicate populations. The threshold of 18.999 mm^2^ was decided based on visual inspection of the size distribution. Estimates are from univariate models.
