# Supplementary material for: Estimation of genetic parameters for the implementation of selective breeding in commercial insect production
Source: Genet Sel Evol. 2024 Mar 25;56:21. doi: 10.1186/s12711-024-00894-7 (PMC10962107; doi:10.1186/s12711-024-00894-7)
Supplement: Supplementary file 4 — Additional file 4: Figure S2. Mating success. Figure illustrating the number of females each male house fly reproduced with. [file 12711_2024_894_MOESM4_ESM.docx]

**Additional file 4 Figure S2: Mating success**

**
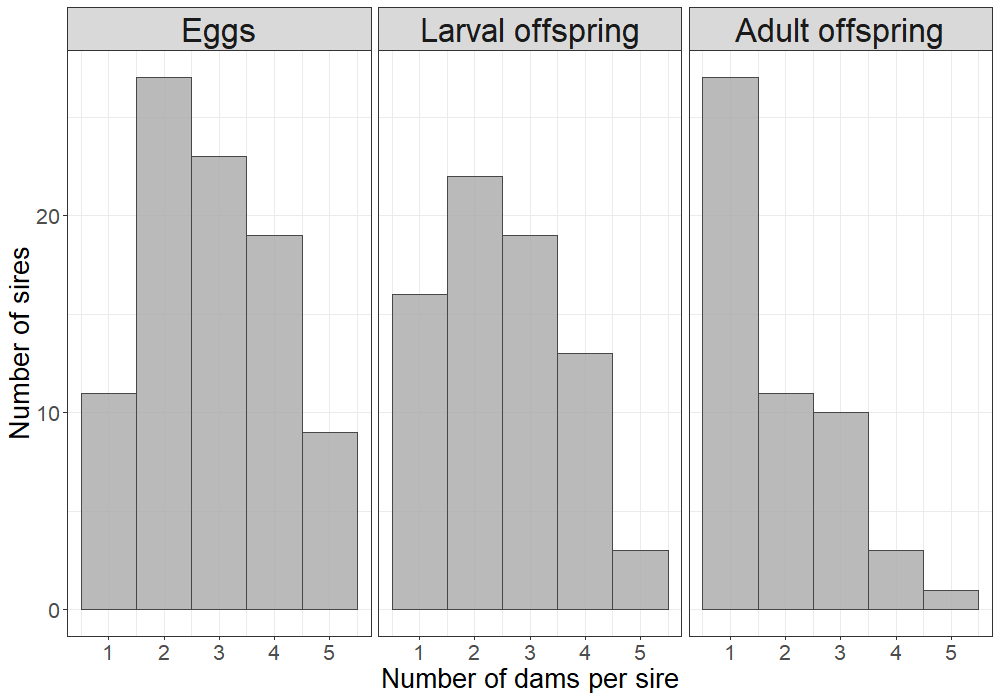
**

**Matings with successful egg-production and offspring with phenotypic records.** The number of sires mating with 1-5 egg-laying dams (left), larval producing dams (middle) and adult producing dams (right). Each sire was exposed to five females for mating, but some females did not lay eggs or produced offspring that were eventually phenotyped for either larval or adult traits. A number of attempted matings resulted in zero egg-laying or offspring producing females, and those matings are not included in the plot.
