# Supplementary material for: Estimation of genetic parameters for the implementation of selective breeding in commercial insect production
Source: Genet Sel Evol. 2024 Mar 25;56:21. doi: 10.1186/s12711-024-00894-7 (PMC10962107; doi:10.1186/s12711-024-00894-7)
Supplement: Supplementary file 5 — Additional file 5: Table S3. Systematic effects. Table of fixed effect estimates from univariate Gaussian models for each of the analysed traits. [file 12711_2024_894_MOESM5_ESM.docx]

**Additional file 5 Table S3: Systematic effects**

| Larval size (mm^2^) | | | | | | | |
| --- | --- | --- | --- | --- | --- | --- | --- |
|  | Levels | N | Empirical BLUE | | SE |  |  |
| Population replicate | Pop A | 638 | 1.19 | 2.94 | | |  |
|  | Pop B | 708 | 0.08 | 2.91 | | |  |
|  | Pop C | 377 | -1.19 | 3.04 | | |  |
| Dam age | 10 days | 1601 | 9.50 | 1.56 | | |  |
|  | 14 days | 122 | 0.00 | 0.00 | |  |  |
| Observer : time-to-oviposition | 1:12 hours | 464 | 9.25 | 2.61 | | |  |
|  | 1:>12 hours | 96 | 1.11 | 2.91 | | |  |
|  | 2:12 hours | 578 | 10.01 | 2.58 | | |  |
|  | 2:>12 hours | 96 | 2.16 | 2.98 | | |  |
|  | 3:12 hours | 256 | 11.06 | 2.73 | | |  |
|  | 3:>12 hours | 52 | 7.09 | 3.95 | | |  |
|  | 4:12 hours | 118 | 9.57 | 3.09 | | |  |
|  | 4:>12 hours | 43 | 0.00 | 0.00 | |  |  |
| Larval survival | | | | | |  |  |
| Population replicate | Pop A | 1480 | -0.04 | 0.10 | |  |  |
|  | Pop B | 1660 | -0.01 | 0.09 | |  |  |
|  | Pop C | 860 | -0.05 | 0.10 | |  |  |
| Dam age | 10 days | 3300 | 0.26 | 0.05 | |  |  |
|  | 14 days | 700 | 0.00 | 0.00 | |  |  |
| Observer : time-to-oviposition | 1:12 hours | 960 | 0.28 | 0.09 | |  |  |
|  | 1:>12 hours | 480 | 0.04 | 0.09 | |  |  |
|  | 2:12 hours | 1200 | 0.29 | 0.09 | |  |  |
|  | 2:>12 hours | 400 | 0.10 | 0.10 | |  |  |
|  | 3:12 hours | 480 | 0.33 | 0.09 | |  |  |
|  | 3:>12 hours | 80 | 0.42 | 0.14 | |  |  |
|  | 4:12 hours | 220 | 0.29 | 0.11 | |  |  |
|  | 4:>12 hours | 180 | 0.00 | 0.00 | |  |  |
| Development time (hours) | | | | | |  |  |
| Population replicate | Pop A | 253 | 483.24 | 15.75 | |  |  |
|  | Pop B | 272 | 500.10 | 15.44 | |  |  |
|  | Pop C | 105 | 500.66 | 18.00 | |  |  |
| Sex | Female | 310 | 9.12 | 2.23 | |  |  |
|  | Male | 320 | 0.00 | 0.00 | |  |  |
| Observer | 1 | 219 | -0.54 | 16.41 | |  |  |
|  | 2 | 241 | 1.24 | 16.40 | |  |  |
|  | 3 | 130 | 13.14 | 17.16 | |  |  |
|  | 4 | 40 | 0.00 | 0.00 | |  |  |
| Adult survival | | | | | |  |  |
| Population replicate | Pop A | 1420 | 0.11 | 0.06 | |  |  |
|  | Pop B | 1640 | 0.10 | 0.06 | |  |  |
|  | Pop C | 840 | 0.05 | 0.07 | |  |  |
| Observer | 1 | 1420 | 0.06 | 0.06 | |  |  |
|  | 2 | 1540 | 0.06 | 0.06 | |  |  |
|  | 3 | 540 | 0.10 | 0.07 | |  |  |
|  | 4 | 400 | 0.00 | 0.00 | |  |  |

**Systematic effects.** Fixed effect parameter estimates (empirical BLUE) with standard errors (SE) from univariate analyses of larval size and survival, egg-to-adult development time and egg-to-adult survival. Fixed effects include population replicate (A, B, C), dam age (10 or 14 days), observer (person counting the eggs, 1-4 where 4 is the sum of the observations from three observers), duration from mating to egg-laying (time-to-oviposition, either 12 or >12 hours) and sex (male or female). Survival traits are binary (0 for dead, 1 for survived). N = number of observations.
